# A Prebiotic Diet Containing Galactooligosaccharides and Polydextrose Produces Dynamic and Reproducible Changes in the Gut Microbial Ecosystem in Male Rats

**DOI:** 10.3390/nu16111790

**Published:** 2024-06-06

**Authors:** Robert S. Thompson, Samuel J. Bowers, Fernando Vargas, Shelby Hopkins, Tel Kelley, Antonio Gonzalez, Christopher A. Lowry, Pieter C. Dorrestein, Martha Hotz Vitaterna, Fred W. Turek, Rob Knight, Kenneth P. Wright, Monika Fleshner

**Affiliations:** 1 Department of Integrative Physiology, University of Colorado Boulder, Boulder, CO 80309, USA; robert.s.thompson@colorado.edu (R.S.T.); tel.kelley@colorado.edu (T.K.); christopher.lowry@colorado.edu (C.A.L.); kenneth.wright@colorado.edu (K.P.W.J.); 2 Center for Neuroscience, University of Colorado Boulder, Boulder, CO 80309, USA; 3 Department of Neurobiology, Northwestern University, Center for Sleep and Circadian Biology, Evanston, IL 60208, USA; bosamuel@med.umich.edu (S.J.B.); m-vitaterna@northwestern.edu (M.H.V.); fturek@northwestern.edu (F.W.T.); 4 Skaggs School of Pharmacy and Pharmaceutical Sciences, University of California San Diego, San Diego, CA 92093, USApdorrestein@health.ucsd.edu (P.C.D.); 5 Department of Pediatrics, University of California San Diego, San Diego, CA 92093, USArknight@ucsd.edu (R.K.); 6 Department of Computer Science and Engineering, University of California San Diego, San Diego, CA 92093, USA; 7 Center for Microbiome Innovation, University of California San Diego, San Diego, CA 92093, USA

**Keywords:** microbiome, metabolome, prebiotic, polydextrose, galactooligosaccharide, *Parabacteroides*, *Ruminiclostridium 5*, bile acid, deoxycholic acid

## Abstract

Despite substantial evidence supporting the efficacy of prebiotics for promoting host health and stress resilience, few experiments present evidence documenting the dynamic changes in microbial ecology and fecal microbially modified metabolites over time. Furthermore, the literature reports a lack of reproducible effects of prebiotics on specific bacteria and bacterial-modified metabolites. The current experiments examined whether consumption of diets enriched in prebiotics (galactooligosaccharides (GOS) and polydextrose (PDX)), compared to a control diet, would consistently impact the gut microbiome and microbially modified bile acids over time and between two research sites. Male Sprague Dawley rats were fed control or prebiotic diets for several weeks, and their gut microbiomes and metabolomes were examined using 16S rRNA gene sequencing and untargeted LC–MS/MS analysis. Dietary prebiotics altered the beta diversity, relative abundance of bacterial genera, and microbially modified bile acids over time. PICRUSt2 analyses identified four inferred functional metabolic pathways modified by the prebiotic diet. Correlational network analyses between inferred metabolic pathways and microbially modified bile acids revealed deoxycholic acid as a potential network hub. All these reported effects were consistent between the two research sites, supporting the conclusion that dietary prebiotics robustly changed the gut microbial ecosystem. Consistent with our previous work demonstrating that GOS/PDX reduces the negative impacts of stressor exposure, we propose that ingesting a diet enriched in prebiotics facilitates the development of a health-promoting gut microbial ecosystem.

## 1. Introduction

The gut microbiome is a diverse ecosystem that consists of bacteria, archaea, eukaryotes, fungi, and viruses that live in the host’s digestive tract [1,2,3]. Microorganisms residing in the digestive tract comprise a micro-ecosystem displaying established principles of ecosystem dynamics [4]. Like any ecosystem, gut microbes both compete and cooperate for limited resources [5]. Dietary macronutrients [6,7] and micronutrients [8] can rapidly change the gut’s microbial composition. Non-digestible complex carbohydrates and types of fermentable fiber, for example, are dietary substrates selectively utilized by host microorganisms that can rapidly alter the gut microbiome and the fecal metabolome and positively impact host health. In 2017, the International Scientific Association for Dietary Probiotics and Prebiotics released a consensus report defining prebiotics as substrates selectively utilized by host microorganisms conferring health benefits [9]. The complex changing dynamics in the gut’s microbial composition after introducing dietary prebiotics have seldom been examined, because doing so requires repeated sampling over time and costly sequencing of large numbers of samples.

Despite substantial evidence supporting the efficacy of prebiotics for promoting host health, there is a paucity of literature replicating prebiotic impacts on bacteria and bacterially modified metabolites over time [10,11,12,13]. The failure to reproduce findings could be due, in part, to multiple bacterial taxonomy databases, ongoing taxonomic revisions, as well as differences in sample storage, DNA extraction and sequencing, and analytic pipelines [14]. In addition, commonly overlooked are the influences of environmental factors on the gut microbiome, including geographic location and elevation [15,16,17,18], and animal source [19,20].

GOS and PDX increase the relative abundance of the bacterial species *Parabacteroides distasonis* and *Clostridium leptum* [21,22], decrease microbially modified secondary bile acids like deoxycholic and lithocholic acid [23,24], and reduce the adverse effects of stress exposure on host sleep physiology [25,26,27]. To make progress towards elucidating the mechanisms for the stress-protective impact on host sleep physiology [27,28], the effects of prebiotics on the gut microbiota and metabolome must be sufficiently robust to resist any potential environmental and methodological influences.

Here, we present the results from two dietary prebiotic animal studies conducted at Northwestern University (NW) in Evanston, Illinois, and the University of Colorado Boulder (CU) in Boulder, Colorado. The two sites have several environmental differences, including different research personnel, vivarium facilities, elevations (182 m vs. 1624 m), and animal sources (Envigo vs. Harlan). To reduce the impact of other factors, NW and CU adhered to standardized fecal sample collection and storage protocols, DNA extraction and sequencing, as well as untargeted LC–MS/MS metabolomics protocols.

The first goal of this project was to determine whether consumption of the same dietary prebiotic formulation tested at different universities, in different locations across the country, and at different times of the year, would produce similar dynamic changes in the gut microbial composition and microbially modified bile acids. The second goal of the study was to explore the potential functional metabolic pathways and networks impacted by the prebiotic diet. We hypothesize that the consumption of GOS/PDX by rats at NW and CU produces robust changes over time in the gut microbiome, fecal metabolome, functional metabolic pathways, and networks.

## 2. Materials and Methods

### 2.1. Animals

Male Sprague Dawley rats were tested. Female rats were not tested in these experiments because this study was supported by funding from the Office of Naval Research (ONR MURI N00014-15-1-2809), and ~80–90% of submariners are male, making males a priority for the ONR’s limited funding. Some data presented here were included in previously published work from the more extensive ONR study, which demonstrated that diets enriched in prebiotics (GOS and PDX) facilitate host sleep/circadian recovery both during and after stressor exposure [21,22].

#### 2.1.1. Northwestern (NW) Study

The animals (*N* = 64, Envigo Laboratories, Madison, WI, USA) were singly housed in a controlled temperature (23 ± 2 °C) and humidity. All the protocols were approved by the Northwestern Institutional Animal Care and Use Committee, as previously described [21]. The animals weighed 40–50 g upon arrival at postnatal day (PND) 23 and were maintained in a 12:12 h light/dark cycle. On arrival, all the rats were housed in Nalgene Plexiglas cages (Thermo Fisher Scientific, Waltham, MA, USA) and were placed on a control or prebiotic diet (ad libitum).

#### 2.1.2. University of Colorado Boulder (CU) Study

The animals (*N* = 82, Harlan Laboratories, Indianapolis, IN, USA) were singly housed with a controlled temperature (23 ± 2 °C) and humidity. All procedures were approved by the University of Colorado Boulder Institutional Animal Care and Use Committee, as previously described [22]. Briefly, the animals weighed 40–50 g upon arrival at PND 23 and were maintained in a 12:12 h light/dark cycle. On arrival, all the rats were housed in Nalgene Plexiglas cages (Thermo Fisher Scientific, Waltham, MA, USA) and were placed on a control or prebiotic diet (ad libitum).

### 2.2. Experimental Design

**Figure 1:**
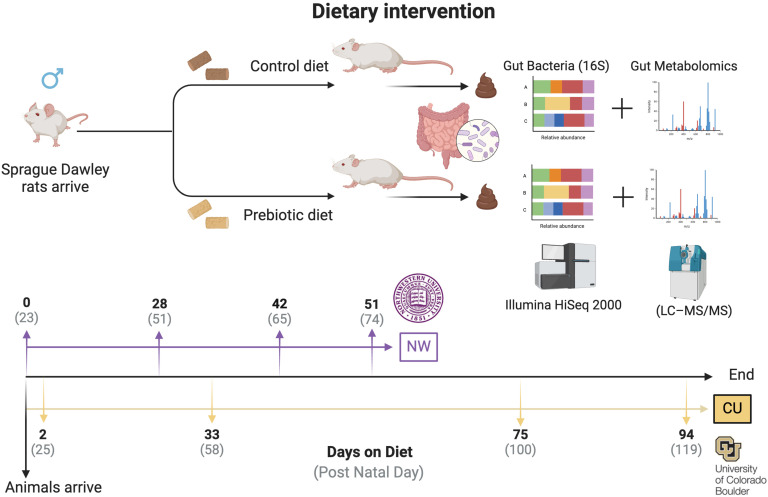
Experimental timeline detailing methods and fecal sampling events. In both studies, animals arrived on postnatal day 23 and were immediately placed on either the control diet or prebiotic diet. In the Northwestern study, fecal samples were taken on experimental (postnatal) days 0 (23), 28 (51), 42 (65), and 51 (74), while in the CU study, fecal samples were taken on experimental days 2 (25), 33 (58), 75 (100), and 94 (119).

The rats arrived at NW on PND 23 and were randomly placed on either the control or prebiotic diet for the duration of the study (Figure 1). Animal numbers for the NW microbiome data were control (*n* = 30) and prebiotic diet (*n* = 32), while the animal numbers for the NW metabolome data were control (*n* = 31) and prebiotic diet (*n* = 32). The rats arrived at CU on PND 23 and were immediately placed on either the control or prebiotic diet for the duration of the study (Figure 1). Animal numbers for the CU microbiome data were control (*n* = 37) and prebiotic diet (*n* = 37), while the animal numbers for the CU metabolome data were control (*n* = 40) and prebiotic diet (*n* = 42). Only samples present for all time points with viable data (i.e., useable fecal samples, high-quality sequencing, quality feature detection, etc.) were included in the final analysis.

At NW, fecal samples were collected on experimental days 0, 28, 42, and 51. At CU, fecal samples were collected on experimental days 2, 33, 75, and 94 (Figure 1). The days chosen for fecal collection differed between the sites due to the other goals of the larger ONR project. The repeated sample collection over time allows one to capture any changes in the microbiome and metabolome due to aging (i.e., adolescence to young adulthood). The two experiments were conducted on Sprague Dawley rats eating identical diets and, thus, give us unique insights into how the gut microbiome and gut metabolome change from adolescence to young adulthood between study sites in response to a prebiotic diet (Figure 1).

### 2.3. Diets

Rats at both facilities had ad libitum access to control or prebiotic diets immediately upon arrival on PND 23. The control and prebiotic diets fed to rats at NW and CU were the same formulation. The diets were initially formulated by Mead Johnson Nutrition (MJN, Evansville, IN, USA) based on AIN-93G specifications, were custom made by Envigo Teklad (TD.110889; now Inotiv, Lafayette, IN, USA), and were isocaloric, with similar carbohydrate, protein, fat, vitamin, and mineral levels, the details of which have been previously published [27,29]. The prebiotic diet contained the following prebiotic substrates, which were absent from the control diet: galactooligosaccharides (GOS, 24.14 g/kg (7.0 g active); FrieslandCampina, Zwolle, The Netherlands), and polydextrose (PDX, 7.69 g/kg (7.0 g active); Danisco, Terre Haute, IN, USA).

### 2.4. Fecal Sample Collection Procedures

Fecal samples were collected and prepared as previously described [30] and were collected after cage change. Sterile forceps (100% ethanol) were used to obtain each sample, which were then placed in 1.5 mL sterile screw cap tubes (USA Scientific, Ocala, FL, USA) and put in liquid nitrogen. The samples were then transferred and stored at −80 °C for analyses later. Weekly fecal samples were collected during the light cycle (~900–1100 h) shortly after cage changes. Investigators collected the rat fecal samples immediately after the rats defecated in the new bedding, i.e., within ~10–30 min. At each collection time point, duplicate samples of bedding, water, food, and blank tubes were also collected to control for potential environmental influences on the microbiome and metabolome data. For both study sites, the fecal samples were cut in half lengthwise to ensure each animal’s microbiome and metabolomics data were generated from the same fecal pellet [31].

### 2.5. The 16S rRNA Gene Sequencing

For both study sites, DNA was extracted from fecal samples and the V4 region of the 16S rRNA gene was amplified using the 515f/806r primer pair with the barcode on the forward read [32], and sequenced as previously described [33]. The samples were purified and precipitated to remove polymerase chain reaction (PCR) artifacts; the samples were sequenced in multiplex using an Illumina HiSeq 2000 (San Diego, CA, USA). All the target gene sequence processing was conducted with Quantitative Insights Into Microbial Ecology (QIIME2) [34] via Qiita. The raw sequencing data were trimmed and demultiplexed at 150 bases. Amplicon sequence variants (ASVs) were generated using the deblur algorithm. Phylogeny was created via SEPP within the QIIME2 fragment insertion plugin, using default parameters. Taxonomy classification was conducted via the QIIME2 feature classifier plugin and based on SILVA [35]. The resulting ASV table was filtered to remove mislabeled samples with a probability above 0.20 using the sample type field, as described in the Human Microbiome Project [36]. The resulting table was then rarefied at 10,000 sequences/sample to correct for an uneven sequencing depth due to amplification differences between the samples. 

Beta diversity was examined with principal coordinate analysis (PCoA) using unweighted UniFrac distances (sensitive to rarer taxa) and weighted UniFrac distances (sensitive to abundances of taxa), which are the best ways to visualize the microbiome between treatments as a whole [37]. For analysis, PERMANOVA was used on each time point in QIIME2. Alpha diversity is a within-samples measure and was examined using evenness, observed OTUs, and Faith’s phylogenetic diversity [38]. Differential abundance was assessed in regard to the ASVs, using analysis of the composition of microbiomes (ANCOM) [39], as implemented in QIIME2 and matched with the SILVA database. Consistent with current recommended best practices [40], we refer to the taxonomy assignments as they are designated in the SILVA database since it is updated annually [35,41], and is based on ASVs, not the construction of molecular operational taxonomic units (OTUs) [40].

PICRUSt2 (https://github.com/picrust/picrust2, accessed on 30 June 2022) was performed in the conda environment for both studies, to identify functionally enriched signaling pathways due to prebiotic diet consumption [42].

The 16S rRNA gene sequencing data were uploaded to Qiita, are publicly available, and can be found at https://qiita.ucsd.edu/study/description/11697 (accessed on 18 November 2021) for the NW study and at https://qiita.ucsd.edu/study/description/11525 (accessed on 18 November 2021) for the CU study.

### 2.6. LC–MS/MS Metabolomics

Fecal and environmental samples were transferred overnight via dry ice to the University of California San Diego and processed for metabolomic analysis. The fecal samples were stored in 1.5 mL centrifuge tubes at −80 °C prior to extraction. Sample IDs were uploaded into an electronic spreadsheet and subsequently used to assign filenames during LC–MS/MS data acquisition. All solvents used for the metabolomic analysis were of LC–MS grade.

This method was adapted from a previously published protocol [43]. Fecal pellets were weighed at 50.0 ± 2 mg wet weight and transferred to 2.0 mL round bottom microcentrifuge tubes (Qiagen Catalog# 990381, Hilden, Germany) for metabolite extraction. A clean stainless-steel bead (Qiagen Catalog# 69989) and 1.5 mL of chilled extraction solvent (50% MeOH) were added to each sample. The samples were then homogenized for 5 min at 25 Hz using a TissueLyser II system (Qiagen Catalog# 85300) and incubated for 20 min at −20 °C. The fecal homogenates were centrifuged at 14,000 rpm for 15 min at 4 °C. Then, 1.2 mL aliquots were transferred into a Nunc 2.0 mL DeepWell plate (Thermo Catalog# 278743) and frozen at −80 °C, before lyophilization using a FreeZone 4.5 L Benchtop Freeze Dryer with Centrivap Concentrator (Labconco, Kansas City, MO, USA). The wells were resuspended with 200 µL of resuspension solvent (50% MeOH spiked with 2.0 µM sulfadimethoxine), vortexed for 30 s, and centrifuged at 2000 rpm for 15 min at 4 °C. Then, 150 µL of the supernatant was transferred into a 96-well plate and maintained at 4 °C, before LC–MS analysis. A resuspension solvent QC and a six standard mix QC (50% MeOH spiked with 1.0 µM sulfamethazine, 1.0 µM sulfamethizole, 1.0 µM sulfachloropyridazine, 1.0 µM amitriptyline, and 1.0 µM coumarin 314) was run every 12th sample to assess the sample background, carry over, chromatography behavior, peak picking, and plate effects.

The fecal extracts were analyzed using an ultra-high performance liquid chromatography system (Vanquish, Thermo Fisher Scientific, Waltham, MA, USA), coupled to a hybrid quadrupole-Orbitrap mass spectrometer (Q-Exactive, Thermo), fitted with a HESI probe. Reverse phase chromatographic separation was achieved using a Kinetex C18 1.7 µm, 100 Å, 50 × 2.1 mm column (Phenomenex, Torrance, CA, USA) held at 40 °C, with a 0.5 mL/min flow rate. Moreover, 5.0 µL aliquots were injected per sample/QC. The mobile phase used was: (A) 0.1% formic acid in water and (B) 0.1% formic acid in acetonitrile. The elution gradient was: 5% B for 1 min, increased to 100% B in the next 8 min, held at 100% B for 2 min, returned to 5.0% B in 0.5 min, and equilibrated at 5.0% B for 2 min. The positive electrospray ionization parameters were: a sheath gas flow rate of 52 (arb. units), an aux gas flow rate of 14 (arb. units), a sweep gas flow rate of 3 (arb. units), a spray voltage of 3.5 kV, a capillary temperature of 270 °C, an S-Lens RF level of 50 (arb. units), and an aux gas heater temperature of 435 °C. The negative electrospray ionization parameters were: a sheath gas flow rate of 52 (arb. units), an aux gas flow rate of 14 (arb. units), a sweep gas flow rate of 3 (arb. units), a spray voltage of 2.5 kV, a capillary temperature of 270 °C, an S-Lens RF level of 50 (arb. units), and an aux gas heater temperature of 435 °C. MS data were acquired using a data dependent acquisition method, with a resolution of 35,000 in MS^1^ and 17,000 in MS^2^. An MS^1^ scan from 100–1500 *m*/*z* was followed by an MS^2^ scan, produced by collision-induced disassociation, of the five most abundant ions from the prior MS^1^ scan.

Feature tables were generated for the control and prebiotic diet samples. To annotate features with a level 1 metabolome standard initiative (MSI) level of confidence, the mass and retention time were aligned and the MS/MS fragmentation pattern was compared between the features and 20 purified bile acid reference standards, as previously described in detail [30,44]. Primary, secondary, conjugated, and unconjugated bile acids were purchased (Cayman Chemical, Ann Arbor, MI, USA) and used to identify level 1 bile acid identification in fecal metabolomics samples. The samples were solubilized to a final concentration of 10 μM in 50% MeOH, before LC–MS/MS injection.

All untargeted mass spectrometry data can be found in the online mass spectrometry repository, Massive (http://massive.ucsd.edu, accessed on 11 May 2022), using the following accession numbers for NW, MSV000083073, and for CU, MSV000080628.

### 2.7. Statistical Analysis

The data were analyzed using R statistics version 4.2.2 GUI 1.79 Big Sur ARM build (8160). The data depicted in the figures were made in Prism (version 9.3.1). For the gut microbiome analysis of the UniFrac distance matrices, permutation multivariate analysis of variance (PERMANOVA) was used at each time point [45,46]. Measures of alpha diversity were analyzed separately using repeated measures ANOVA. To investigate differential abundance of genera level taxa between the control and prebiotic diets, a first-level analysis of the composition of the microbiome (ANCOM) was performed on the ASVs [39] to reveal reliable changes. ANCOM analysis will correct for multiple comparisons of ASVs identified in the sequencing data. The ASVs that were undefined/unclassified at the genera level were excluded from the final analysis. Once the taxonomy was assigned, we performed a second level of analysis on genus-level taxonomy assignments using the Nonparametric Tests for Repeated Measures Data in Factorial Designs (nparLD) package version 2.2. Importantly, only genera that were significantly changed by the prebiotic diet based on the ANCOM analysis are presented in this manuscript. Lower relative abundance genera were non-normally distributed; therefore, these data were analyzed using the nparLD package. The bile acid data were log transformed, as previously described [22,44], and analyzed using the nparLD package. Multiple significant *p*-values in the bile acid data were adjusted using the Holm method. The pathways output from PICRUSt2 was analyzed via DESeq2 version 1.14.1, using the Bioconductor R package, as previously described [21], and volcano plot analysis by the time point. The pathways affected by the prebiotic diet between the study sites and over time were analyzed using nparLD. Tukey’s post hoc analysis was used when appropriate using the nparcomp, the nonparametric relative contrast effects (nparcomp) package version 3.0, for relative abundance of the genera, bile acids, and pathway data. Network analyses examining the relationships between functionally significant pathways and bile acids were performed using the corrr package version 0.4.4. The two-tailed alpha level was set at *p* < 0.05.

## 3. Results

### 3.1. Microbiome

**Figure 2:**
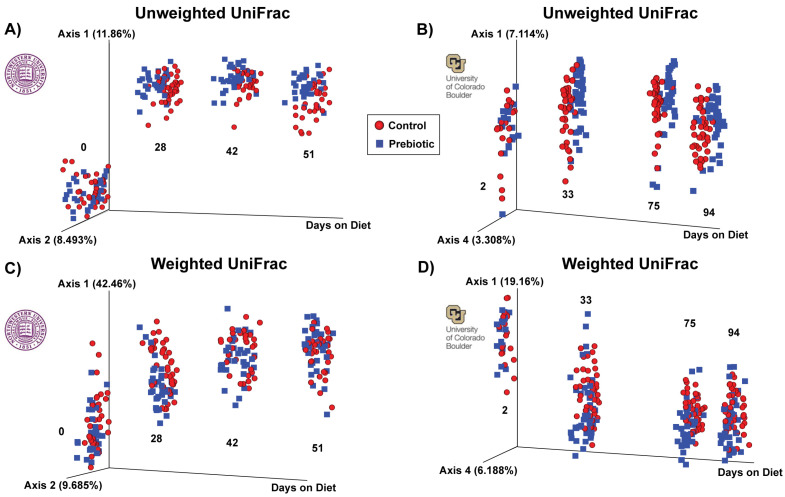
Unweighted and weighted UniFrac distance examining β-diversity of the fecal microbiome between studies. (**A**) In the NW study, unweighted UniFrac distance at experimental day 0 was not different between the control and prebiotic diets, but was different on subsequent days 28, 42, and 51. (**B**) In the CU study, unweighted UniFrac distance at experimental day 2 was not different between the control and prebiotic diets, but was different on subsequent days 33, 75, and 94. (**C**) In the NW study, weighted UniFrac distance was not different on day 0 between the control and prebiotic diets, but was different on the remaining days examined. (**D**) In the CU study, weighted UniFrac distance was significantly different on day 2 between the control and prebiotic diets, an effect that persisted for days 33, 75, and 94.

A prebiotic diet significantly changed the beta diversity of the gut microbiome at both study sites (Figure 2). Table 1 denotes the significant effects of a prebiotic diet on weighted and unweighted UniFrac distances. The prebiotic diet had no effect at 0 days on the diet on the weighted or unweighted UniFrac distance (NW). There was a significant effect of the prebiotic diet at 2 days on the diet on the weighted UniFrac distance (CU). The prebiotic diet significantly impacted both the weighted and unweighted UniFrac distance on all the remaining days on the diet at both study sites (Figure 2, Table 1).

The prebiotic diet altered the two main phyla (Firmicutes and Bacteroidetes) in the rat gut microbiome (Supplemental Figure S1). The main significant main effects of the prebiotic diet were on the Firmicutes at NW (*F*_(1,2.77)_ = 9.72; *p* = 0.002; Supplemental Figure S1A) and CU (*F*_(1,2.85)_ = 20.94; *p* = 0.0000078; Supplemental Figure S1A). The effect of the prebiotic diet changed over time at NW (time-by-diet interaction, *F*_(1,59.99)_ = 4.61; *p* = 0.004), but not CU. The prebiotic diet also impacted the Bacteroidetes at both NW (*F*_(1,2.73)_ = 6.01; *p* = 0.014; Supplemental Figure S1B) and CU (*F*_(1,2.83)_ = 20.94; *p* = 0.0000047; Supplemental Figure S1B). Finally, there were time-by-diet interactions in regard to the Bacteroidetes that changed over time at both NW (*F*_(1,59.76)_ = 3.33; *p* = 0.022; Supplemental Figure S1B) and CU (*F*_(1,71.01)_ = 3.39; *p* = 0.019; Supplemental Figure S1B). While these phyla changes are important, it is of more interest to examine the taxonomic changes in greater detail at the genera level.

**Figure 3:**
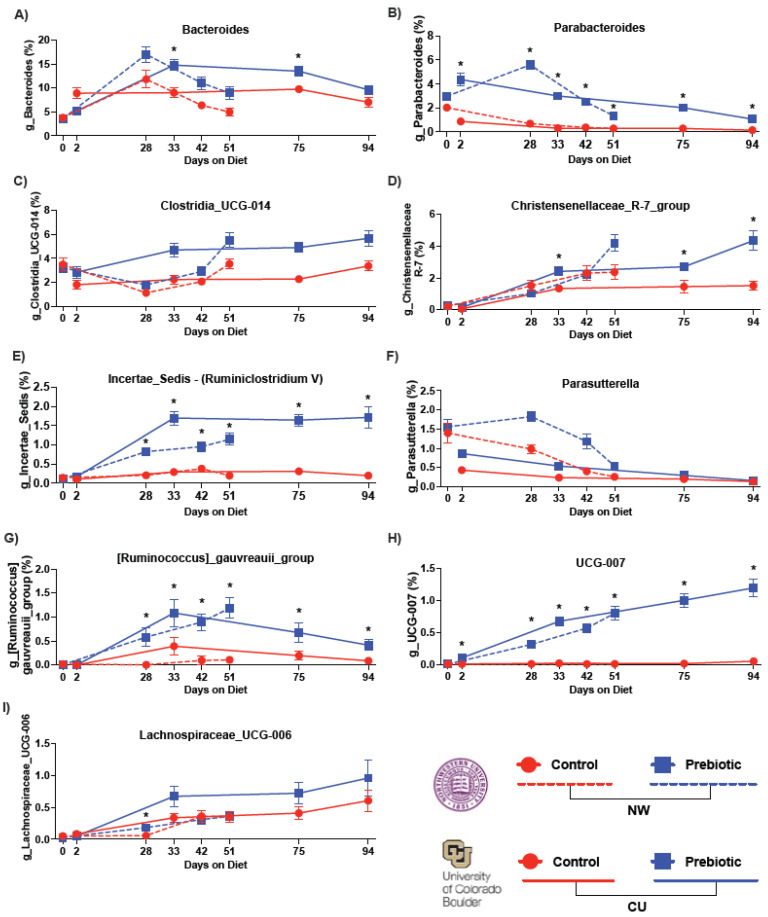
Consumption of the prebiotic diet produced increases in 9 higher abundance genera between studies. There were consistent increases over time due to the prebiotic diet in: (**A**) *Bacteroides*, (**B**) *Parabacteroides*, (**E**) *Incertae_Sedis* (*Ruminiclostridium V*), (**G**) *Ruminococcus_gauvreauii_group*, and (**H**) *UCG-007*. While there were prebiotic diet-induced increases in (**C**) *Clostridia_UCG-014*, (**D**) *Christensenellaceae_R-7_group*, (**F**) *Parasutterella*, and (**I**) *Lachnospiraceae_UCG-006*, these genera had less consistent increases over time between studies. * *p* < 0.05 when compared to control diet.

The top nine most abundant genera increased by the prebiotic diet, when compared to the control diet, are shown in Figure 3. The prebiotic diet increased the relative abundance of the genera *Bacteroides* (Figure 3A), *Parabacteroides* (Figure 3B), *Clostridia_UCG_014* (Figure 3C), *Incertae_Sedis* (formerly known as *Ruminiclostridium V*) (Figure 3E), *Parasutterella* (Figure 3F), *Ruminococcus_gauvreauii_group* (Figure 3G), and *UCG-007* (Figure 3H), at both study sites. See Table 2 and Figure 3 for complete statistical and post hoc analysis results. There were differing effects between study sites in regard to *Christensenellaceae_R-7_group* (Figure 3D) and *Lachnospiraceae_UCG-006* (Figure 3I), such that the prebiotic diet increased these genera in the CU study, but not in the NW study. However, there was a diet-by-time interaction in regard to *Lachnospiraceae_UCG-006* at NW (see Figure 3I for the results of the post hoc analysis).

**Figure 4:**
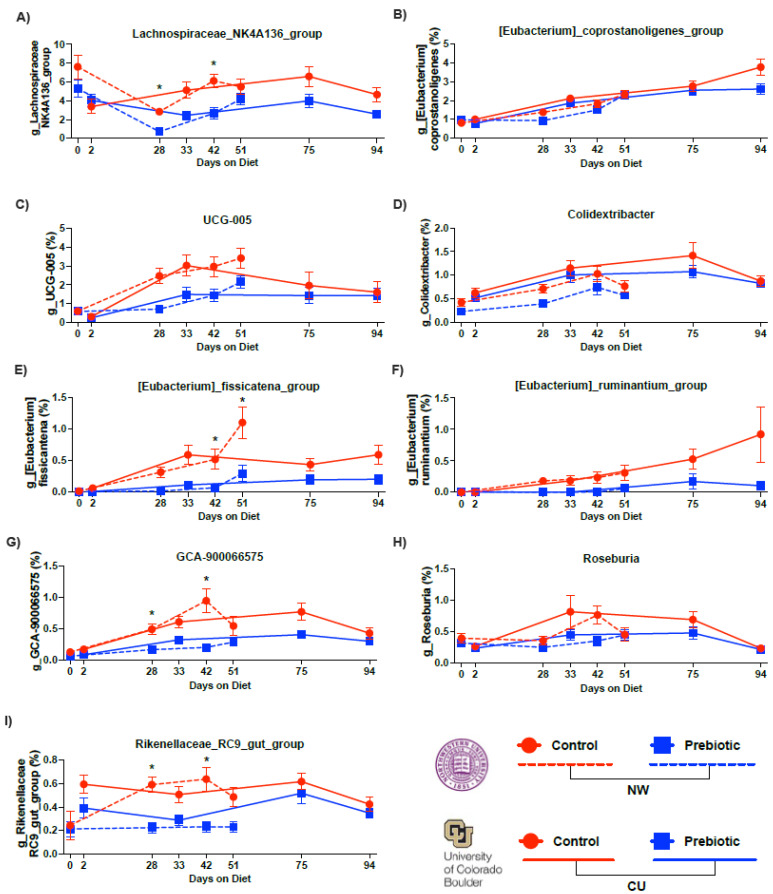
Consumption of prebiotic diet led to decreases in 6 higher abundance genera between studies. There were consistent decreases over time due to prebiotic diet consumption in: (**A**) *Lachnospiraceae_NK4A136_group*, (**C**) *UCG-005*, (**E**) *Eubacterium_fissicatena_group*, (**F**) *Eubacterium_ruminantium_group*, (**G**) *GCA-900066575*, and (**I**) *Rikenellaceae_R9-gut_group*. There were less consistent effects due to diet between studies in: (**B**) *Eubacterium_ coprostanoligenes_group*, (**D**) *Colidextribacter*, and (**H**) *Roseburia*. * *p* < 0.05 when compared to prebiotic diet.

The top six (out of nine) most abundant genera that were consistently lower in the prebiotic diet group, when compared to the control diet group, are shown in Figure 4. *Lachnospiraceae_NK4A136_group* (Figure 4A), *Eubacterium_fissicatena_group* (Figure 4E), *Eubacterium_ruminantium_group* (Figure 4F), *GCA-900066575* (Figure 4G), *Rosburia* (Figure 4H), and *Rikenellaceae_RC9_gut_group* (Figure 4I) were consistently lower in the prebiotic diet group, when compared to the control diet groups, at each study site (see Table 2 for the statistical analysis; see Figure 4 for the results of the post hoc tests). *Colidextribacter* (Figure 4D) and *UCG-005* (Figure 4C) were lower in the prebiotic diet group in the NW study, but not in the CU study (Table 2). The prebiotic diet had no significant effects on *Eubacterium_coprostanoligenes_group* at either study site (Figure 4B).

Supplemental Figure S2 depicts the additional ultra-low abundance genera (less than 1% relative abundance). Supplemental Table S1 contains the corresponding statistics. Overall, the impact of a prebiotic diet on ultra-low relative abundance (<1%) genera was less consistent between the study sites, except for the genus *Tuzzerella*. *Tuzzerella* was lower in the prebiotic diet groups at both study sites (Supplemental Figure S2, Supplemental Table S1). Finally, the prebiotic diet impacted six genera with opposite effects between study sites, although these were not statistically significant once corrected via Tukey’s post hoc analysis (Supplemental Figure S3, Supplemental Table S2). One interesting finding was the difference in the relative abundance in the genus *Muribaculaceae* between the study sites. The relative abundance of this genus was higher in the NW versus the CU microbiome data. This large inherent environmental difference present in the genus *Muribaculaceae* may have played a role in the contrasting effects of the prebiotic diet in the alpha diversity results between the study sites.

**Figure 5:**
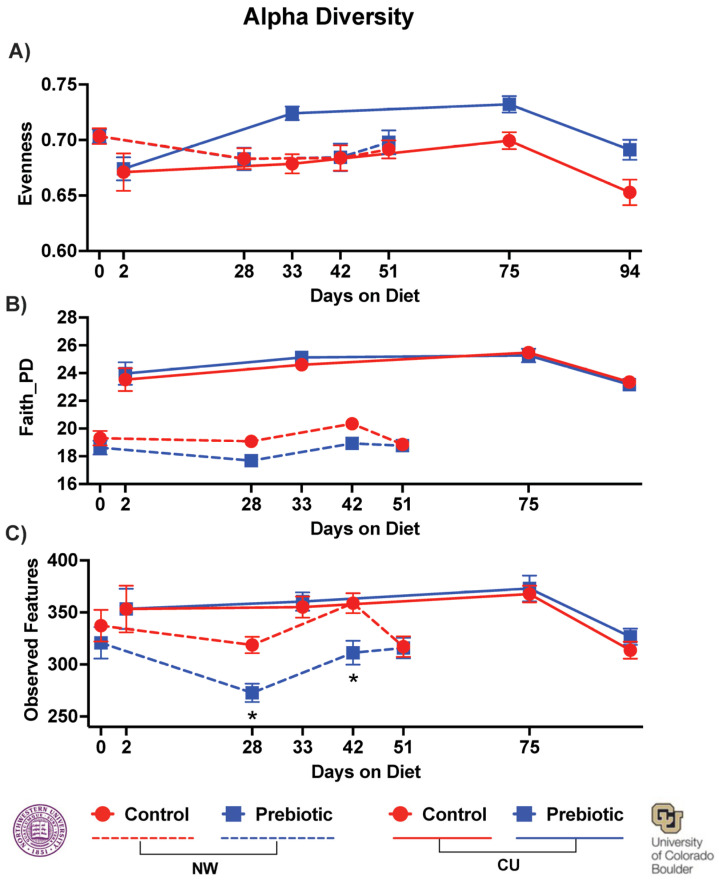
There was a significant main effect of the prebiotic diet, increasing (**A**) the evenness of the alpha diversity in the CU study. In contrast, the main significant effects of the prebiotic diet involved decreases in both (**B**) Faith’s phylogenetic diversity and (**C**) the observed features of the alpha diversity in the NW study. There were no significant time-by-diet interactions in regard to the measures of alpha diversity, except at NW in observed features. * *p* < 0.05 effect of diet.

Dietary prebiotics inconsistently altered the alpha diversity at both study sites. In the NW study, there was no effect of the prebiotic diet on evenness (Figure 5A); however, in the CU study, the prebiotic diet increased the overall evenness (*F*_(1,3)_ = 12.27; *p* = 0.00084; Figure 5A). In the NW study, there was a significant decrease in Faith’s phylogenetic diversity due to the prebiotic diet (*F*_(1,3)_ = 5.82; *p* = 0.021), while no effects were found due to the prebiotic diet in Faith’s phylogenetic diversity in the CU study (Figure 5B). The observed features were lower due to the prebiotic diet at NW (*F*_(1,3)_ = 6.25; *p* = 0.017), and there was a significant diet-by-time interaction (*F*_(1,3)_ = 2.95; *p* = 0.035), but the prebiotic diet did not affect the observed features in the CU study (Figure 5C).

### 3.2. Metabolome—Bile Acids

**Figure 6:**
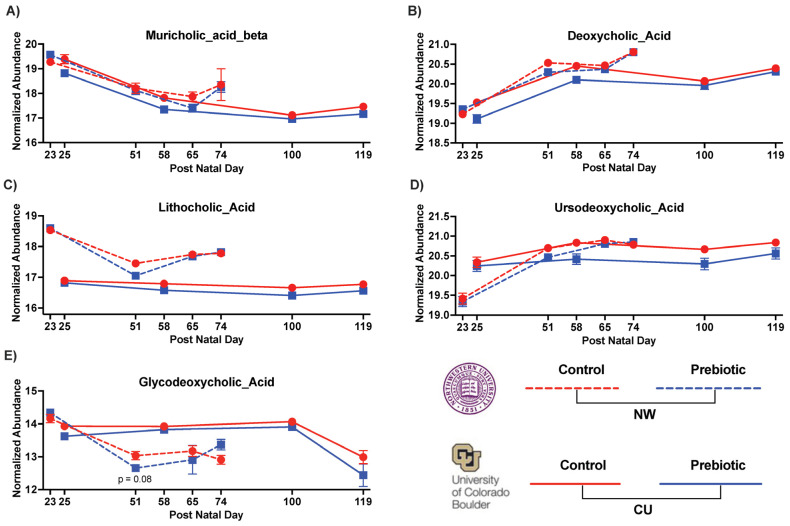
Consumption of dietary prebiotics affected fecal bile acids between studies, including: (**A**) muricholic acid beta, (**B**) deoxycholic acid, and (**C**) lithocholic acid. Moreover, (**D**) ursodeoxycholic acid was decreased in the CU study, and (**E**) glycodeoxycholic acid was decreased in the NW study.

Overall, the relative abundance of several bile acids was lower in the prebiotic diet groups compared to the control diet groups and these results were directionally consistent across the study sites (Figure 6; see Table 3 with statistics for all bile acids identified). Specifically, the primary bile acid muricholic beta was lower in the prebiotic diet groups in both studies (Figure 6A; significant main effect at CU, significant interaction at NW). The secondary bile acids, deoxycholic acid (Figure 6B; significant main effects) and lithocholic acid (Figure 6C; significant main effect at CU, significant interaction at NW), were lower in the prebiotic diet groups between the study sites. In the CU study, ursodeoxycholic acid was also lower in the prebiotic diet group (significant main effect) but was unaffected in the NW study (Figure 6D; see Table 3). Finally, the secondary conjugated bile acid, glycodeoxycholic acid, was impacted by the prebiotic diet in the NW study and unaffected in the CU study (see Figure 6E for the results of the post hoc analyses). Table 3 lists the bile acids not affected by the prebiotic diet when corrected for multiple comparisons.

### 3.3. PICRUSt2—Pathways

**Figure 7:**
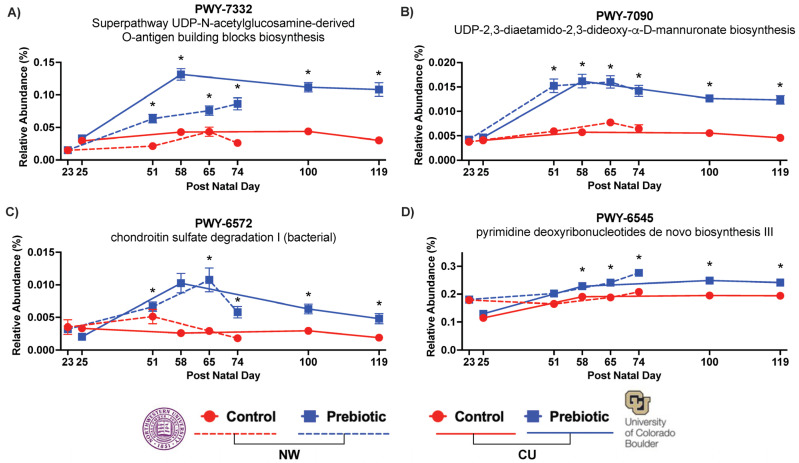
Functional metabolic pathways affected by prebiotic diet, annotated with the MetaCyc metabolic pathway database. Consumption of dietary prebiotics altered the: (**A**) superpathway UDP-N-acetylglucosamin-derived O-antigen building blocks biosynthesis (PWY-7332) or the UDP-sugar superpathway, the (**B**) UDP-2,3-diaetamido-2,3-dideoxy-α-D-mannuronate biosynthesis (PWY-7090) or UDP mannuronate pathway, the (**C**) chondroitin sulfate degradation I (bacterial) pathway (PWY-6572), and the (**D**) pyrimidine deoxyribonucleotides de novo biosynthesis III pathway (PWY-6545), when compared to the control diet. These effects were consistent between the study sites and over time. * *p* < 0.05 when compared to control diet.

In both studies, the prebiotic diet consistently affected the inferred functional metabolic pathways PWY-7332, PWY-7090, PWY-6572, and PWY-6545 over time (Figure 7). The superpathway UDP-N-acetylglucosamine-derived O-antigen building blocks biosynthesis or PWY-7332 was significantly higher in the prebiotic diet in the NW study (*F*_(2.71,59.49)_ = 60.04; *p* < 0.0001; Figure 7A) and in the CU study (*F*_(1,2.62)_ = 182.60; *p* < 0.0001; Figure 7A). The UDP-2,3-diaetamido-2,3-dideoxy-α-D-mannuronate biosynthesis or PWY-7090 was significantly higher in the prebiotic diet in the NW study (*F*_(1,2.81)_ = 71.76; *p* < 0.0001; Figure 7B) and in the CU study (*F*_(1,2.47)_ = 132.21; *p* < 0.0001; Figure 7B). The chondroitin sulfate degradation I (bacterial) or PWY-6572 was also significantly higher in the prebiotic diet in the NW study (*F*_(1,2.79)_ = 56.76; *p* < 0.0001; Figure 7C) and in the CU study (*F*_(1,2.80)_ = 43.33; *p* < 0.0001; Figure 7C). Finally, the prebiotic diet increased the pyrimidine deoxyribonucleotides de novo biosynthesis III or PWY-6545 in the NW study (*F*_(1,2.87)_ = 17.29; *p* < 0.0001; Figure 7D) and in the CU study (*F*_(1,2.74)_ = 30.10; *p* < 0.0001; Figure 7D). There was also significant diet-by-time interactions for PWY-7332 in the NW study (*F*_(2.71,59.49)_ = 11.90; *p* < 0.0001; see Figure 7A for the results of the post hoc comparisons) and the CU study (*F*_(2.62,69.98)_ = 16.90; *p* < 0.0001; see Figure 7A for the results of the post hoc comparisons); for PWY-7090 in the NW study (*F*_(2.81,57.58)_ = 9.13; *p* < 0.0001; see Figure 7B for the results of the post hoc comparisons) and the CU study (*F*_(2.47,62.56)_ = 15.61; *p* < 0.0001; see Figure 7B for the results of the post hoc comparisons); for PWY-6572 in the NW study (*F*_(2.79,58.32)_ = 4.05; *p* = 0.008; see Figure 7C for the results of the post hoc comparisons) and the CU study (*F*_(2.80,67.23)_ = 12.36; *p* < 0.0001; see Figure 7C for the results of the post hoc comparisons); and for PWY-6545 in the NW study (*F*_(2.87,57.55)_ = 3.47; *p* = 0.017; see Figure 7D for the results of the post hoc comparisons) and the CU study (*F*_(2.74,69.77)_ = 16.90; *p* < 0.0001; see Figure 7D for the results of the post hoc comparisons).

### 3.4. Correlation Network Analysis

**Figure 8:**
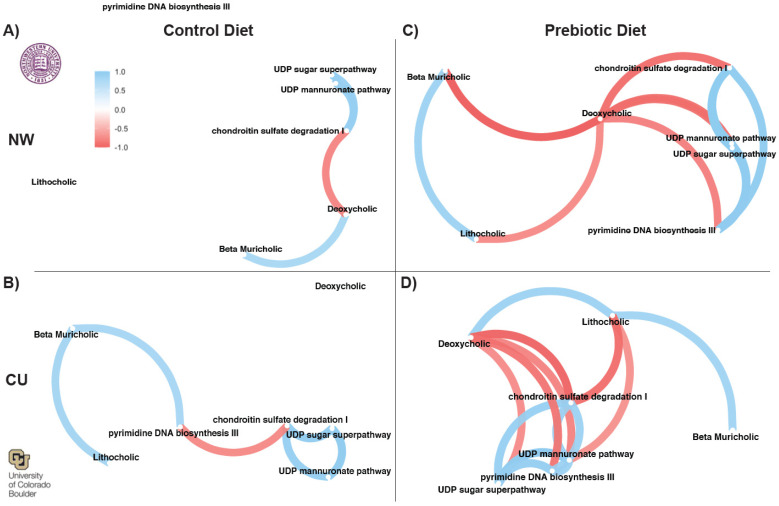
Network correlations from both study sites, demonstrating consistent networks between inferred functional metabolic pathways and bile acids in prebiotic diet groups. There were no consistent correlation networks present in the control diet groups between the study sites (**A**,**B**). The consistent correlation networks in the prebiotic diet groups (**C**) at NW and (**D**) at CU imply that the microbially modified secondary bile acid, deoxycholic acid, could be an important component underlying the beneficial effects of dietary prebiotics.

Correlation network analysis was performed to examine the similarities in the prebiotic diet effects between the study sites with output from the network analyses, as shown in Figure 8. The input into the networks were bile acids (Figure 6) and inferred pathways (Figure 7), which were significantly affected by the prebiotic diets between the study sites. There were no consistent correlations between the pathways and bile acids in the control diets across the studies (Figure 8A,B). In contrast, there were consistent correlation networks between the inferred pathways and the bile acid data in the prebiotic diet groups (Figure 8C,D). The prebiotic diet groups had consistent negative correlations between deoxycholic acid and the four inferred pathways (Figure 8). There was also a consistent positive correlation between lithocholic acid and beta muricholic acid beta between the study sites in the prebiotic diet groups. One difference, however, was a negative correlation between deoxycholic acid and lithocholic acid at NW (Figure 8C), but a positive correlation between these two bile acids at the CU study site (Figure 8D).

## 4. Discussion

The ingestion of a diet enriched in GOS/PDX produces dynamic and robust changes in the gut microbial composition and microbially dependent bile acids. Despite differences in research personnel, animal facilities, geographic locations, elevations, and animal sources, the temporal pattern of changes in the microbial community structure, microbially dependent metabolites, and functional metabolic pathways, was replicated between the study sites. The prebiotic diet also modulated the relative abundance of several genera, reduced microbially modified bile acids, and altered the networks between inferred functional microbial pathways and microbially modified gut bile acids. Importantly, these changes were sufficiently robust to overcome potential environmental differences between the studies.

Based on measures of β-diversity (UniFrac distance), which take into account phylogenetic relationships [47], dietary prebiotics changed both the weighted and unweighted UniFrac distance at both study sites. In the CU study, the weighted UniFrac distance was altered after 2 days on a prebiotic diet, suggesting the rapid growth of higher abundance genera. Dietary prebiotics produced significant compositional changes in the α-diversity metrics (evenness, Faith’s phylogenetic diversity, observed features) at both study sites; however, the metrics of the induced changes were different. In the NW study, prebiotics reduced Faith’s phylogenetic diversity and observed species, whereas in the CU study, prebiotics increased the evenness. These variable impacts of a prebiotic diet on α-diversity between the study sites could reflect inherent differences in the starting microbiomes between the study sites.

The consumption of a diet enriched in GOS/PDX at NW and CU increased the relative abundance of the *Bacteroides* genus. Based on the ASV and prior shotgun sequencing data from a subset of these samples, *Bacteroides uniformis*, a member of the *Bacteroides* genus, was also significantly increased (*p* = 0.0003) by GOS/PDX [22]. The ingestion of *Bacteroides uniformis* produces metabolic, immune, and exercise endurance benefits [48,49]. These studies support the idea that an increased relative abundance of specific taxa within the *Bacteroides* genus may be health promoting.

The consumption of a diet enriched in GOS/PDX also increased the relative abundance of the *Parabacteroides* genus. The *Parabacteroides* genus has been shown to be decreased with a high-fat diet and increased with exercise [50]. *Parabacteroides distasonis* is a species within the *Parabacteroides* genus. GOS/PDX supplementation increases *Parabacteroides distasonis* and restores disturbed sleep and circadian rhythm [21,22]. Based on these studies, increases in the relative abundance of specific taxa within the *Parabacteroides* genus may be health promoting. Importantly, however, *Parabacteroides* growth left unchecked or not kept in balance could be detrimental to the gut microbial ecosystem [51].

Additional changes to the gut microbial ecosystem include prebiotic-induced increases in the specific taxa within the genera *Incertae_Sedis* (formerly known as *Ruminiclostridium V* based on ASV) and the *Ruminococcus gauvreauii group.* Increases in the levels of *Ruminiclostridium V* subsequent to the administration of PDX are associated with improved cognitive performance [52]. And, in contrast, low levels of *Ruminiclostridium V* have been reported for people with kidney stones [53] and rats with acute necrotizing pancreatitis [54]. Consistent with our data, the genera *Ruminococcus gauvreauii group* is increased by fructooligosaccharides [24], and this genus is lower in individuals with obesity [55], coronary artery disease [56] and Parkinson’s disease [57]. These findings taken together, therefore, suggest that the genera *Incertae_Sedis* (formerly known as *Ruminiclostridium V* based on ASV) and *Ruminococcus gauvreauii group* may be health promoting.

The genus *UCG-007* was also increased over time similarly between studies, but little is known about it other than that it varies seasonally [58]. The genera *Clostridia_UCG-014*, *Christensenellaceae_R-7_group*, *Parasutterella*, and *Lachnospiraceae_UCG-006* were also all elevated due to the prebiotic diet, but the temporal effects on these genera were less consistent between the study sites.

In addition to increases in the relative abundance of health-promoting genera, several genera were reduced by the prebiotic diet. Most notably, the genus *Lachnospiraceae_NK4A136_group* was consistently lower in the prebiotic diet groups at both study sites and has recently been implicated in gut mucous membrane function [59]. The genus *UCG-005*, within the Oscillospiraceae family, was lower in the prebiotic diet groups. This lower relative abundance of *UCG-005* may be health promoting given that *UCG-005* is elevated in diabetes patients and is associated with elevated uric acid [60]. The genus *Eubacterium_fissicatena_group* was lower in the prebiotic diet groups and is potentially harmful to bone mineral density [61] and correlates with obesity in a high-fat diet model [62]. The prebiotic diet also lowered *Eubacterium_ruminantium_group*, *GCA-900066575*, and *Rikenellaceae_RC9_gut_group*. Less is known about how and if these genera are related to host health.

Not only did prebiotics change the microbial composition of the gut microbiome, but they also impacted specific features of the gut metabolome. The sequencing data were analyzed using PICRUSt2 and annotated with the MetaCyc metabolic pathway database. These analyses identified four inferred functional metabolic pathways that were changed by the prebiotic diet. Importantly, the prebiotic diet impacted the same pathways between the study sites, with remarkably similar time courses. The first pathway, the UDP-sugar superpathway (PWY-7332), is involved in building the O-antigen polysaccharide for gram-negative bacteria, including *Parabacteroides distasonis*, which is a component of lipopolysaccharide. The second pathway, the UDP mannuronate biosynthesis pathway (PWY-7090), was identified for both study sites and is involved in UDP-sugar metabolism. Clearly, the consumption of dietary prebiotics affected the UDP-sugar pathway. The third pathway affected by prebiotics, chondroitin sulfate degradation I (PWY-6572), is involved in the degradation of chondroitin sulfate, which is a sulfated glycosaminoglycan that can affect the gut microbiome composition [63] and increase fecal butyrate levels in stressed mice [64]. The fourth pathway was pyrimidine DNA biosynthesis III (PWY-6545), which is involved in the biosynthesis of the activated precursors of DNA/RNA.

While the significance of how the gut metabolome and host physiology are affected by changes in these inferred pathways cannot be deduced from the PICRUSt2 analysis, there is evidence that consumption of GOS/PDX facilitated host sleep/circadian recovery after stressor exposure [21,22]. Clearly, the consumption of GOS/PDX consistently affected these four functional metabolic pathways between the study sites similarly over time. These findings support the idea that dietary prebiotics consistently and similarly altered the micro-ecosystem of the gut microbiome.

A prebiotic diet changes specific gut metabolites with bioactive potential, including microbially modified secondary bile acids [21,22]. Prebiotic diet consumption produced similar decreases in fecal deoxycholic acid and lithocholic acid between the study sites. It has been demonstrated that the consumption of a diet enriched in isomaltulose [23] and fructooligosaccharide [24] prebiotics also reduces fecal lithocholic and deoxycholic acid. In contrast, a high-fat diet increases both fecal deoxycholic acid and intestinal inflammation [65]. Here, we report that GOS/PDX reduces fecal deoxycholic acid and lithocholic acid, and this finding was consistent at both study sites. The current data and prior studies support the conclusion that the consumption of a prebiotic diet reduces fecal bile acids and changes the micro-ecosystem of the gut, similarly.

Our findings indicate that the consumption of a prebiotic diet consistently affects functional metabolic pathways and fecal bile acid profiles. We conducted network correlation analyses between functional metabolic pathways and fecal bile acids to determine whether these changes are related. Correlational networks between pathways and bile acids were not observed in the control diet groups. However, network correlations were found in the prebiotic groups. Specifically, bile acids were significantly correlated with the functional metabolic pathways. The network correlations in both the prebiotic diet groups were remarkably similar between the study sites, with what appears to be a network hub related to deoxycholic acid. Based on these findings and previous work, we hypothesize that decreases in deoxycholic acid may be a key metabolic feature underlying the potential health-promoting effects of GOS/PDX. Deoxycholic acid can bind to the Takeda G protein-coupled receptor 5 (TGR5), which is specific to bile acids and is known to activate several intracellular signaling pathways [66,67].

## 5. Conclusions

We demonstrate that dietary GOS/PDX produces robust and reproducible changes in the microbial composition of the gut micro-ecosystem, sufficient to overcome unforeseen environmental impacts, addressing a gap in the literature [10,11,12,13]. Although some variations between the NW study and the CU study exist, the consistent pattern of taxonomic changes over time and impacts on functional metabolic pathways are similar. We identified consistent correlational networks associating the changes in bile acids and functional pathways, which supports the robust nature of the effects. Notably, the networks were found in the prebiotic groups and not the control diet groups, supporting the conclusion that the changes are driven by prebiotics. Finally, these key findings were reproduced at both study sites. Overall, a prebiotic diet increases and decreases the relative abundance of several genera, which may support a health-promoting gut micro-ecosystem.

## Figures and Tables

**Table 1:** PERMANOVA table demonstrating significant effects of prebiotic diet by time point at both study sites. Numbers represent days on the diet.

PERMANOVAs (Pseudo-F)				
	Northwestern			
	0	28	42	51
Unweighted	*F*_(2,68)_ = 1.24; *p* = 0.154	*F*_(2,80)_ = 7.68; *p* = 0.001	*F*_(2,66)_ = 5.60; *p* = 0.001	*F*_(2,69)_ = 5.87; *p* = 0.001
Weighted	*F*_(2,68)_ = 2.19; *p* = 0.053	*F*_(2,80)_ = 9.31; *p* = 0.001	*F*_(2,66)_ = 4.26; *p* = 0.001	*F*_(2,66)_ = 4.34; *p* = 0.001
	University of Colorado Boulder			
	2	33	75	94
Unweighted	*F*_(2,48)_ = 1.31; *p* = 0.053	*F*_(2,78)_ = 4.89; *p* = 0.001	*F*_(2,83)_ = 3.84; *p* = 0.001	*F*_(2,84)_ = 4.16; *p* = 0.001
Weighted	*F*_(2,48)_ = 3.97; *p* = 0.006	*F*_(2,78)_ = 10.99; *p* = 0.001	*F*_(2,83)_ = 7.39; *p* = 0.001	*F*_(2,84)_ = 3.93; *p* = 0.001

**Table 2:** Nonparametric longitudinal data (nparLD) table: ANOVA-type statistics (ATSs) showing the similar significant effects of a prebiotic diet over time on genera identified through ANCOM, between study sites.

Nonparametric Longitudinal Data (nparLD) Table: ANOVA-Type Statistics (ATSs)							
Genera (Relative Abundance)							
		Diet—*F*-Value; *p*-Value		Time—*F*-Value; *p*-Value		Diet × Time	
Higher in Prebiotic Diet, color indicates consistent effect across study site							
High Relative Abundance (2–20%)							
Bacteroides (Figure 3A)	NW	*F*_(1,2.74)_ = 12.62	*p* = 0.00038	*F*_(2.74,59.91)_ = 41.26	*p* = 1.47 × 10^24^	*F*_(2.74,59.91)_ = 2.78	*p* = 0.044
	CU	*F*_(1,2.76)_ = 7.42	*p* = 0.0064	*F*_(2.76,70.42)_ = 17.71	*p* = 9.39 × 10^11^	*F*_(2.76,70.42)_ = 9.90	*p* = 3.63 × 10^6^
Clostridia_UCG-014 (Figure 3C)	NW	*F*_(1,2.65)_ = 4.72	*p* = 0.029	*F*_(2.65,55.97)_ = 38.09	*p* = 4.85 × 10^22^	*F*_(2.65,55.97)_ = 1.01	*p* = 0.379
	CU	*F*_(1,2.71)_ = 35.93	*p* = 2.05 × 10^9^	*F*_(2.71,67.05)_ = 12.88	*p* = 8.44 × 10^8^	*F*_(2.71,67.05)_ = 2.18	*p* = 0.095
Christensenellaceae_R7_group (Figure 3D)	NW	*F*_(1,2.82)_ = 3.70	*p* = 0.054	*F*_(2.82,53.48)_ = 79.63	*p* = 1.30 × 10^48^	*F*_(2.82,53.48)_ = 2.59	*p* = 0.055
	CU	*F*_(1,2.81)_ = 48.65	*p* = 3.06 × 10^12^	*F*_(2.81,69.23)_ = 197.98	*p* = 2.63 × 10^120^	*F*_(2.81,69.23)_ = 9.16	*p* = 8.53 × 10^6^
Incertae_Sedis (Ruminiclostridium V) (Figure 3E)	NW	*F*_(1,2.81)_ = 76.70	*p* = 1.99 × 10^18^	*F*_(2.81,56.25)_ = 46.03	*p* = 4.62 × 10^28^	*F*_(2.81,56.25)_ = 16.85	*p* = 2.09 × 10^10^
	CU	*F*_(1,2.51)_ = 210.79	*p* = 9.26 × 10^48^	*F*_(2.51,71.88)_ = 85.26	*p* = 1.03 × 10^46^	*F*_(2.51,71.88)_ = 19.26	*p* = 7.86 × 10^11^
Parabacteroides (Figure 3B)	NW	*F*_(1,2.74)_ = 158.1	*p* = 2.96 × 10^36^	*F*_(2.74,59.18)_ = 44.69	*p* = 1.32 × 10^26^	*F*_(2.74,59.18)_ = 21.04	*p* = 1.19 × 10^12^
	CU	*F*_(1,2.74)_ = 467.75	*p* = 9.91 × 10^104^	*F*_(2.74,71.88)_ = 71.88	*p* = 6.98 × 10^32^	*F*_(2.74,71.88)_ = 4.99	*p* = 0.00258
Low Relative Abundance (1–2%)							
Parasutterella (Figure 3F)	NW	*F*_(1,78)_ = 29.19	*p* =6.57 × 10^8^	*F*_(2.78,59.45)_ = 40.76	*p* = 1.46 × 10^24^	*F*_(2.78,59.45)_ = 1.09	*p* = 0.127
	CU	*F*_(1,2.78)_ = 9.15	*p* = 0.0025	*F*_(2.78,71.92)_ = 63.79	*p* = 1.78 × 10^38^	*F*_(2.78,71.92)_ = 2.25	*p* = 0.052
Ruminococcus_gauvreauii_group (Figure 3G)	NW	*F*_(1,2.71)_ = 104.03	*p* = 1.99 × 10^24^	*F*_(2.71,59.31)_ = 27.24	*p* = 3.73 × 10^16^	*F*_(2.71,59.31)_ = 17.95	*p* = 9.61 × 10^11^
	CU	*F*_(1,2.33)_ = 16.93	*p* = 0.000039	*F*_(2.33,69.45)_ = 19.51	*p* = 2.48 × 10^10^	*F*_(2.33,69.45)_ = 4.79	*p* = 0.00018
UCG-007 (Figure 3H)	NW	*F*_(1,2.84)_ = 289.83	*p* = 5.42 × 10^65^	*F*_(2.84,55.73)_ = 40.50	*p* = 7.18 × 10^25^	*F*_(2.84,55.73)_ = 31.13	*p* = 3.74 × 10^19^
	CU	*F*_(1,2.66)_ = 140.28	*p* = 2.31 × 10^32^	*F*_(2.66,57.78)_ = 32.24	*p* = 9.19 × 10^19^	*F*_(2.66,57.78)_ = 10.11	*p* = 3.89 × 10^6^
Lachnospiraceae_UCG-006 (Figure 3I)	NW	*F*_(1,2.77)_ = 1.76	*p* = 0.184	*F*_(2.77,59.93)_ = 42.81	*p* = 9.83 × 10^26^	*F*_(2.77,59.93)_ = 4.89	*p* = 0.0028
	CU	*F*_(1,2.77)_ = 6.33	*p* = 0.0118	*F*_(2.77,65.94)_ = 51.97	*p* = 3.48 × 10^31^	*F*_(2.77,65.94)_ = 4.61	*p* = 0.00410
Higher in Control Diet, color indicates consistent effect across study site							
High Relative Abundance (2–20%)							
Lachnospiraceae_NK4A136_group (Figure 4A)	NW	*F*_(1,2.81)_ = 36.70	*p* = 1.38 × 10^9^	*F*_(2.81,59.99)_ = 20.53	*p* = 1.34 × 10^12^	*F*_(2.81,59.99)_ = 2.81	*p* = 0.020
	CU	*F*_(1,2.83)_ = 13.13	*p* = 0.0003	*F*_(2.83,71.45)_ = 2.30	*p* = 0.079	*F*_(2.83,71.45)_ = 3.99	*p* = 0.0087
Eubacterium_coprostanoligenes_group (Figure 4B)	NW	*F*_(1,2.48)_ = 1.34	*p* = 0.247	*F*_(2.48,57.50)_ = 30.14	*p* = 1.56 × 10^16^	*F*_(2.48,57.50)_ = 1.66	*p* = 0.183
	CU	*F*_(1,2.87)_ = 3.64	*p* = 0.056	*F*_(2.87,71.52)_ = 56.35	*p* = 5.72 × 10^35^	*F*_(2.87,71.52)_ = 0.55	*p* = 0.638
UCG-005 (Figure 4C)	NW	*F*_(1,2.82)_ = 8.07	*p* = 0.0451	*F*_(2.82,59.10)_ = 21.62	*p* = 2.59 × 10^13^	*F*_(2.82,59.10)_ = 1.62	*p* = 0.184
	CU	*F*_(1,2.37)_ = 0.841	*p* = 0.359	*F*_(2.37,71.81)_ = 18.66	*p* = 4.57 × 10^10^	*F*_(2.37,71.81)_ = 1.73	*p* = 0.171
Low Relative Abundance (1–2%)							
Colidextribacter (Figure 4D)	NW	*F*_(1,2.73)_ = 13.95	*p* = 0.00019	*F*_(2.73,59.62)_ = 18.55	*p* = 3.78 × 10^11^	*F*_(2.73,59.62)_ = 0.816	*p* = 0.475
	CU	*F*_(1,2.78)_ = 0.013	*p* = 0.911	*F*_(2.78,71.96)_ = 32.26	*p* = 1.64 × 10^8^	*F*_(2.78,71.96)_ = 0.328	*p* = 0.790
Eubacterium_fissicatena_group (Figure 4E)	NW	*F*_(1,2.41)_ = 9.51	*p* = 0.002	*F*_(2.41,54.79)_ = 25.73	*p* = 7.31 × 10^14^	*F*_(2.41,54.79)_ = 7.08	*p* = 0.00034
	CU	*F*_(1,2.26)_ = 4.64	*p* = 0.031	*F*_(2.26,68.41)_ = 18.53	*p* = 1.32 × 10^9^	*F*_(2.26,68.41)_ = 3.09	*p* = 0.039
Eubacterium_ruminantium_group (Figure 4F)	NW	*F*_(1,2.62)_ = 17.80	*p* = 0.00002	*F*_(2.62,38.97)_ = 7.97	*p* = 0.00006	*F*_(2.62,38.97)_ = 11.57	*p* = 6.82 × 10^7^
	CU	*F*_(1,2.44)_ = 6.22	*p* = 0.013	*F*_(2.44,63.83)_ = 8.63	*p* = 0.00005	*F*_(2.44,63.83)_ = 6.31	*p* = 0.0008
GCA-900066575 (Figure 4G)	NW	*F*_(1,2.92)_ = 20.93	*p* = 0.000005	*F*_(2.92,58.16)_ = 24.09	*p* = 3.24 × 10^15^	*F*_(2.92,58.16)_ = 5.18	*p* = 0.0016
	CU	*F*_(1,2.91)_ = 9.67	*p* = 0.0019	*F*_(2.91,71.91)_ = 29.78	*p* = 49.98 × 10^19^	*F*_(2.91,71.91)_ = 0.937	*p* = 0.420
Roseburia (Figure 4H)	NW	*F*_(1,2.80)_ = 6.48	*p* = 0.0109	*F*_(2.80,59.66)_ = 2.90	*p* = 0.037	*F*_(2.80,59.66)_ = 1.73	*p* = 0.161
	CU	*F*_(1,2.74)_ = 4.79	*p* = 0.029	*F*_(2.74,72.00)_ = 8.71	*p* = 0.000019	*F*_(2.74,72.00)_ = 0.776	*p* = 0.50
Rikenellaceae_RC9_gut_group (Figure 4I)	NW	*F*_(1,2.74)_ = 25.70	*p* = 3.99 × 10^7^	*F*_(2.74,59.55)_ = 9.20	*p* = 0.00006	*F*_(2.74,59.55)_ = 5.55	*p* = 0.0012
	CU	*F*_(1,2.72)_ = 10.90	*p* = 0.00096	*F*_(2.72,70.925)_ = 2.88	*p* = 0.040	*F*_(2.72,70.925)_ = 1.42	*p* = 0.236

**Table 3:** Nonparametric longitudinal data (nparLD) table: ANOVA-type statistics (ATSs) showing significant effects of a prebiotic diet over time on all identified bile acids, between study sites.

Nonparametric Longitudinal Data (naprLD) Table: ANOVA-Type Statistics (ATSs)										
Bile Acids										
		Diet—*F*-Value; *p*-Value		*p*-adj. (Holm)	Time—*F*-Value; *p*-Value		*p*-adj. (Holm)	Diet × Time		*p*-adj. (Holm)
Color indicates consistent effect across study site										
Primary Bile Acids										
Cholic Acid	NW	*F*_(1,2.846)_ = 0.190	*p* = 0.663	n/a	*F*_(2.846,58.12)_ = 9.534	*p* = 4.493 × 10^6^	*p* = 8.98 × 10^6^	*F*_(2.846,58.12)_ = 0.921	*p* = 0.426	ns
	CU	*F*_(1,2.605)_ = 4.0759	*p* = *0.0435*	*p* = 0.136	*F*_(2.605,36.68)_ = 4.934	*p* = 0.0033	*p* = 0.0066	*F*_(2.605,36.68)_ = 0.2756	*p* = 0.815	n/a
Muricholic_alpha	NW	*F*_(1,2.72)_ = 0.188	*p* = 0.665	n/a	*F*_(2.72,58.508)_ = 35.817	*p* = 3.20 × 10^21^	*p* = 1.92 × 10^20^	*F*_(2.72,58.508)_ = 0.755	*p* = 0.507	ns
	CU	*F*_(1,2.81)_ = 2.24	*p* = 0.135	ns	*F*_(2.81,77.408)_ = 57.30	*p* = 7.157 × 10^35^	*p* = 1.0024 × 10^33^	*F*_(2.81,77.408)_ = 0.397	*p* = 0.742	n/a
Muricholic_beta (Figure 6A)	NW	*F*_(1,2.911)_ = 2.623	*p* = 0.105	n/a	*F*_(2.911,54.878,)_ = 36.129	*p* = 1.011 × 10^22^	*p* = 8.08 × 10^22^	*F*_(2.911,54.878,)_ = 2.706	*p* = *0.0453*	*p* = 0.0453
	CU	*F*_(1,2.68)_ = 9.452	*p* = *0.0021*	*p* = 0.019	*F*_(2.68,78.32)_ = 81.99	*p* = 1.00 × 10^47^	*p* = 1.9 × 10^46^	*F*_(2.68,78.32)_ = 0.272	*p* = 0.823	n/a
Conjugated Bile Acids										
Glycochenodeoxycholic Acid	NW	*F*_(1,2.819)_ = 0.578	*p* = 0.447	n/a	*F*_(2.819,60.355)_ = 59.90	*p* = 1.503 × 10^36^	*p* = 1.95 × 10^35^	*F*_(2.819,60.355)_ = 1.784	*p* = 0.151	ns
	CU	*F*_(1,2.917)_ = 2.459	*p* = 0.116	ns	*F*_(2.917,78.25)_ = 17.47	*p* = 4.35 × 10^11^	*p* = 3.48 × 10^10^	*F*_(2.917,78.25)_ = 0.508	*p* = 0.671	n/a
Glycocholic Acid	NW	*F*_(1,2.627)_ = 0.146	*p* = 0.701	n/a	*F*_(2.627,60.03)_ = 108.142	*p* = 1.084 × 10^61^	*p* = 1.728 × 10^60^	*F*_(2.627,60.03)_ = 0.257	*p* = 0.831	ns
	CU	*F*_(1,2.744)_ = 4.479	*p* = *0.0343*	*p* = 0.136	*F*_(2.744,75.63)_ = 41.22	*p* = 1.38 × 10^24^	*p* = 1.794 × 10^23^	*F*_(2.744,75.63)_ = 0.109	*p* = 0.274	n/a
Glycohyocholic Acid	NW	*F*_(1,2.913)_ = 0.092	*p* = 0.762	n/a	*F*_(2.913,60.943)_ = 28.238	*p* = 8.523 × 10^18^	*p* = 4.26 × 10^17^	*F*_(2.913,60.943)_ = 0.514	*p* = 0.667	ns
	CU	*F*_(1,2.706)_ = 0.543	*p* = 0.4611	ns	*F*_(2.706,79.81)_ = 29.44	*p* = 2.083 × 10^17^	*p* = 1.872 × 10^16^	*F*_(2.706,79.81)_ = 0.146	*p* = 0.917	n/a
Taurochenodeoxycholic Acid	NW	*F*_(1,2.57)_ = 0.453	*p* = 0.501	n/a	*F*_(2.57,58.52)_ = 43.784	*p* = 1.290 × 10^24^	*p* = 1.161 × 10^23^	*F*_(2.57,58.52)_ = 1.378	*p* = 0.250	ns
	CU	*F*_(1,2.82)_ = 3.133	*p* = 0.0688	ns	*F*_(2.82,76.881)_ = 34.930	*p* = 2.46 × 10^21^	*p* = 2.706 × 10^20^	*F*_(2.82,76.881)_ = 0.567	*p* = 0.625	n/a
Taurocholic Acid	NW	*F*_(1,2.83)_ = 0.417	*p* = 0.518	n/a	*F*_(2.83,60.603)_ = 15.834	*p* = 7.724 × 10^10^	*p* = 2.316 × 10^9^	*F*_(2.83,60.603)_ = 0.743	*p* = 0.519	ns
	CU	*F*_(1,2.773)_ = 6.388	*p* = *0.0115*	*p* = 0.069	*F*_(2.773,36.7228)_ = 1.232	*p* = 0.296	ns	*F*_(2.773,36.7228)_ = 0.644	*p* = 0.575	n/a
Taurohyocholic Acid	NW	*F*_(1,2.581)_ = 2.896	*p* = 0.0878	n/a	*F*_(2.581,58.664)_ = 57.948	*p* = 1.301 × 10^32^	*p* = 1.56 × 10^31^	*F*_(2.581,58.664)_ = 1.256	*p* = 0.288	ns
	CU	*F*_(1,2.381)_ = 4.492	*p* = *0.0341*	ns	*F*_(2.381,79.613)_ = 42.977	*p* = 1.39 × 10^22^	*p* = 1.668 × 10^21^	*F*_(2.381,79.613)_ = 1.348	*p* = 0.259	n/a
Secondary Bile Acids										
Deoxycholic Acid (Figure 6B)	NW	*F*_(1,2.2670)_ = 5.557	*p* = 0.0184	n/a	*F*_(2.2.267,28.994)_ = 84.80	*p* = 3.18 × 10^42^	*p* = 4.77 × 10^41^	*F*_(2.267,28.994)_ = 2.19	*p* = 0.104	ns
	CU	*F*_(1,2.79)_ = 12.219	*p* = *0.00047*	*p* = 0.005	*F*_(2.79,79.83)_ = 62.44	*p* = 8.56 × 10^38^	*p* = 1.3696 × 10^36^	*F*_(2.79,79.83)_ = 2.188	*p* = 0.0918	n/a
Lithocholic Acid (Figure 6C)	NW	*F*_(1,2.832)_ = 0.240	*p* = 0.624	n/a	*F*_(2.832,60.296)_ = 123.84	*p* = 6.77 × 10^76^	*p* = 1.2186 × 10^74^	*F*_(2.832,60.296)_ = 3.374	*p* = *0.0196*	*p* = 0.0392
	CU	*F*_(1,2.90)_ = 10.84	*p* = *0.0009*	*p* = 0.010	*F*_(2.90,79.89)_ = 12.19	*p* = 8.72 × 10^8^	*p* = 3.49 × 10^7^	*F*_(2.90,79.89)_ = 1.476	*p* = 0.220	n/a
Ursodeoxycholic Acid (Figure 6D)	NW	*F*_(1,2.539)_ = 2.465	*p* = 0.164	n/a	*F*_(2.539,60.446)_ = 115.64	*p* = 7.468 × 10^64^	*p* = 1.2699 × 10^62^	*F*_(2.539,60.446)_ = 2.228	*p* = 0.0935	ns
	CU	*F*_(1,2.532)_ = 9.188	*p* = *0.00243*	*p* = 0.019	*F*_(2.532,78.672)_ = 4.966	*p* = 0.00349	*p* = 0.0066	*F*_(2.532,78.672)_ = 1.098	*p* = 0.343	n/a
Secondary Conjugated Bile Acids										
Glycodeoxycholic Acid (Figure 6E)	NW	*F*_(1,2.818)_ = 0.485	*p* = 0.486	n/a	*F*_(2.818,60.903)_ = 48.193	*p* = 2.045 × 10^29^	*p* = 2.255 × 10^28^	*F*_(2.818,60.903)_ = 5.310	*p* = *0.0015*	*p* = 0.0045
	CU	*F*_(1,2.916)_ = 5.013	*p* = *0.0252*	*p* = 0.126	*F*_(2.916,79.05)_ = 31.25	*p* = 1.064 × 10^19^	*p* = 1.06 × 10^18^	*F*_(2.916,79.05)_ = 0.972	*p* = 0.403	n/a
Glycolithocholic Acid	NW	*F*_(1,2.513)_ = 1.268	*p* = 0.260	n/a	*F*_(2.513,59.330)_ = 72.0	*p* = 1.784 × 10^39^	*p* = 2.492 × 10^38^	*F*_(2.513,59.330)_ = 0.534	*p* = 0.627	ns
	CU	*F*_(1,2.513)_ = 0.009	*p* = 0.923	ns	*F*_(2.513,78.64)_ = 76.44	*p* = 6.81 × 10^42^	*p* = 1.1577 × 10^40^	*F*_(2.513,78.64)_ = 0.150	*p* = 0.903	n/a
Glycoursodeoxycholic Acid	NW	*F*_(1,2.488)_ = 0.898	*p* = 0.343	n/a	*F*_(2.488,56.613)_ = 48.827	*p* = 1.26 × 10^26^	*p* = 1.26 × 10^25^	*F*_(2.488,56.613)_ = 0.194	*p* = 0.920	ns
	CU	*F*_(1,2.911)_ = 1.24	*p* = 0.265	ns	*F*_(2.911,79.71)_ = 74.60	*p* = 6.64 × 10^47^	*p* = 1.1952 × 10^45^	*F*_(2.911,79.71)_ = 0.214	*p* = 0.881	n/a
Taurodeoxycholic Acid	NW	*F*_(1,2.501)_ = 0.262	*p* = 0.609	n/a	*F*_(2.501,59.216)_ = 40.489	*p* = 3.021 × 10^22^	*p* = 2.114 × 10^21^	*F*_(2.501,59.216)_ = 0.835	*p* = 0.456	ns
	CU	*F*_(1,2.768)_ = 4.484	*p* = *0.0342*	ns	*F*_(2.768,78.280)_ = 16.797	*p* = 3.07 × 10^10^	*p* = 1.84 × 10^9^	*F*_(2.768,78.280)_ = 0.511	*p* = 0.659	n/a
Taurohyodeoxycholic Acid	NW	*F*_(1,2.817)_ = 1.459	*p* = 0.227	n/a	*F*_(2.817,60.123)_ = 150.64	*p* = 7.138 × 10^92^	*p* = 1.4994 × 10^90^	*F*_(2.817,60.123)_ = 1.111	*p* = 0.341	ns
	CU	*F*_(1,2.785)_ = 7.212	*p* = *0.00724*	*p* = 0.050	*F*_(2.785,79.746)_ = 13.787	*p* = 1.68 × 10^8^	*p* = 8.40 × 10^8^	*F*_(2.785,79.746)_ = 0.473	*p* = 0.687	n/a
Taurolithocholic Acid	NW	*F*_(1,2.895)_ = 0.001	*p* = 0.974	n/a	*F*_(2.895,60.534)_ = 18.164	*p* = 1.887 × 10^11^	*p* = 7.56 × 10^11^	*F*_(2.895,60.534)_ = 2.139	*p* = 0.095	ns
	CU	*F*_(1,2.85)_ = 0.006	*p* = 0.937	ns	*F*_(2.85,78.972)_ = 17.603	*p* = 5.79 × 10^11^	*p* = 4.05 × 10^10^	*F*_(2.85,78.972)_ = 0.784	*p* = 0.497	n/a

## Data Availability

Data are contained within the article and Supplementary Materials.

## Supplementary Materials

The following supporting information can be downloaded at: https://www.mdpi.com/article/10.3390/nu16111790/s1, Figure S1: Phylum-level relative abundance data demonstrating consistent effects of prebiotic diet on the two main phyla: Firmicutes and Bacteroidetes. (A) There was a significant main effect of prebiotic diet on Firmicutes at NW (*F*_(1,2.77)_ = 9.72; *p* = 0.002) and a significant time-by-diet interaction (*F*_(1,59.99)_ = 4.61; *p* = 0.004). There was also a significant main effect of prebiotic diet on Firmicutes at CU (*F*_(1,2.85)_ = 20.94; *p* = 0.0000078) but there was not a significant time-by-diet interaction. (B) There was also a significant main effect of prebiotic diet at on Bacteroidetes at NW (*F*_(1,2.73)_ = 6.01; *p* = 0.014) and a significant time-by-diet interaction (*F*_(1,59.76)_ = 3.33; *p* = 0.022). At CU, there was also a significant main effect of prebiotic diet on Bacteroidetes (*F*_(1,2.83)_ = 20.94; *p* = 0.0000047) and there was a significant time-by-diet interaction (*F*_(1,71.01)_ = 3.39; *p* = 0.019). Notably, in both studies, the relative abundance of Firmicutes increased and Bacteroidetes decreased across time regardless of diet. * *p* < 0.05 when compared to control diet; Figure S2: Data demonstrating the effects of dietary prebiotics on lower abundance genera between study sites across time. * *p* < 0.05 when compared to control diet; Figure S3: Data demonstrating inconsistent effects on six different genera between study sites across time. * *p* < 0.05 when compared to control diet; Table S1: PERMANOVA table demonstrating significant effects of prebiotic diet by time point at both study sites. Numbers represent days on diet; Table S2: Nonparametric longitudinal data (nparLD) Table: ANOVA-type statistic (ATS) showing the similar significant effects of a prebiotic diet across time on genera identified through ANCOM between study sites.
