# Supplementary material for: A Prebiotic Diet Containing Galactooligosaccharides and Polydextrose Produces Dynamic and Reproducible Changes in the Gut Microbial Ecosystem in Male Rats
Source: Nutrients. 2024 Jun 6;16(11):1790. doi: 10.3390/nu16111790 (PMC11175065; doi:10.3390/nu16111790)
Supplement: Supplementary file 1 [file nutrients-16-01790-s001.zip › Figure S2.pdf]

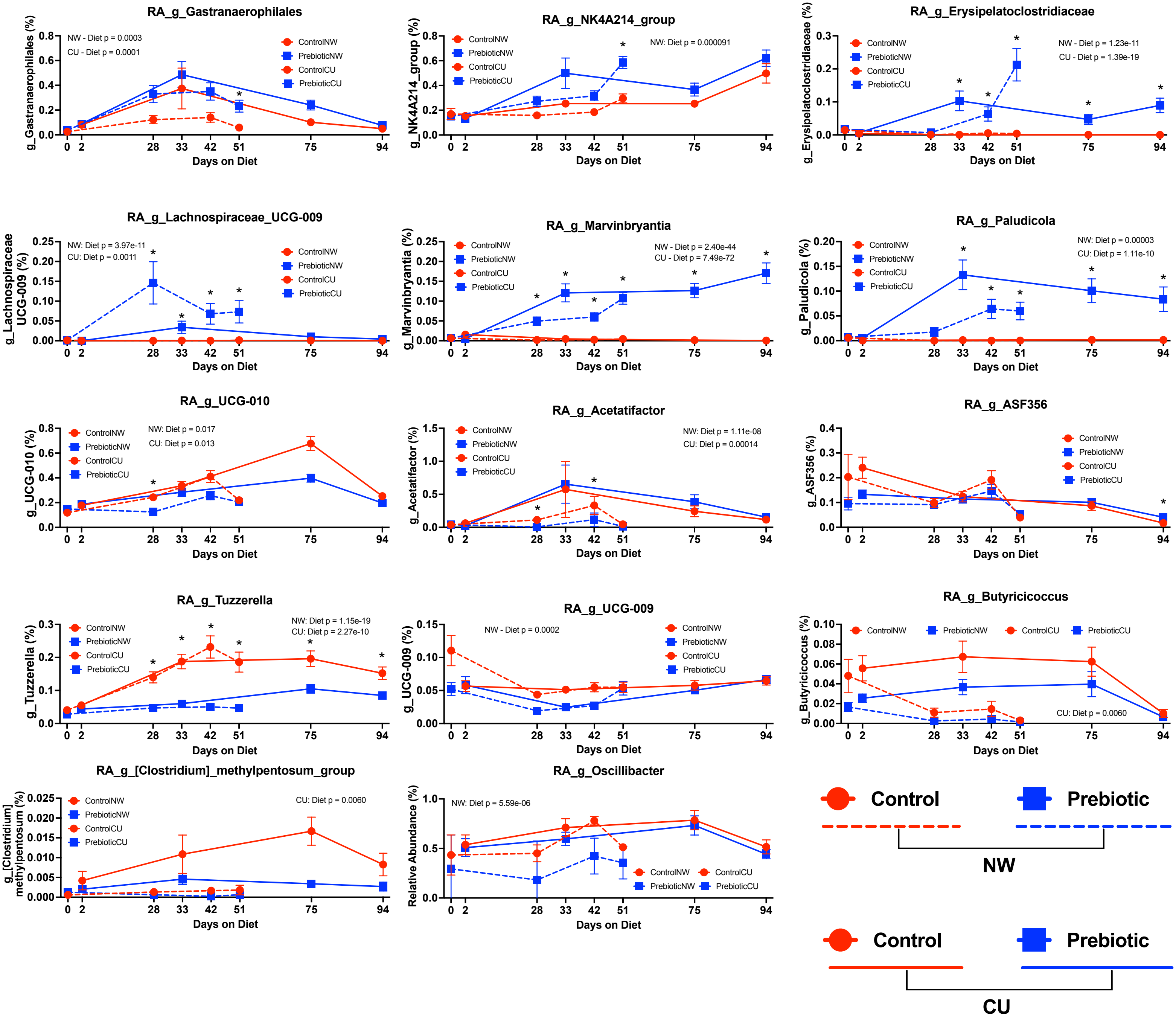
RA_g_Gastranaerophilales
NW - Diet p = 0.0003
CU - Diet p = 0.0001
ControlNW
PrebioticNW
ControlCU
PrebioticCU
g_Gastranaerophilales (%)
Days on Diet
RA_g_NK4A214_group
NW: Diet p = 0.000091
g_NK4A214_group (%)
RA_g_Erysipelatoclostridiaceae
NW - Diet p = 1.23e-11
CU - Diet p = 1.39e-19
g_Erysipelatoclostridiaceae (%)
RA_g_Lachnospiraceae_UCG-009
NW: Diet p = 3.97e-11
CU: Diet p = 0.0011
g_Lachnospiraceae UCG-009 (%)
RA_g_Marvinbryantia
NW - Diet p = 2.40e-44
CU - Diet p = 7.49e-72
g_Marvinbryantia (%)
RA_g_Paludicola
NW: Diet p = 0.00003
CU: Diet p = 1.11e-10
g_Paludicola (%)
RA_g_UCG-010
NW: Diet p = 0.017
CU: Diet p = 0.013
g_UCG-010 (%)
RA_g_Acetatifactor
NW: Diet p = 1.11e-08
CU: Diet p = 0.00014
g_Acetatifactor (%)
RA_g_ASF356
g_ASF356 (%)
RA_g_Tuzzerella
NW: Diet p = 1.15e-19
CU: Diet p = 2.27e-10
g_Tuzzerella (%)
RA_g_UCG-009
NW - Diet p = 0.0002
g_UCG-009 (%)
RA_g_Butyricicoccus
CU: Diet p = 0.0060
g_Butyricicoccus (%)
RA_g_[Clostridium]_methylpentosum_group
CU: Diet p = 0.0060
g_[Clostridium] methylpentosum (%)
RA_g_Oscillibacter
NW: Diet p = 5.59e-06
Relative Abundance (%)
Control
Prebiotic
NW
Control
Prebiotic
CU
