# Supplementary figures and images for: A Prebiotic Diet Containing Galactooligosaccharides and Polydextrose Produces Dynamic and Reproducible Changes in the Gut Microbial Ecosystem in Male Rats
Source: Nutrients. 2024 Jun 6;16(11):1790. doi: 10.3390/nu16111790 (PMC11175065; doi:10.3390/nu16111790)

A)

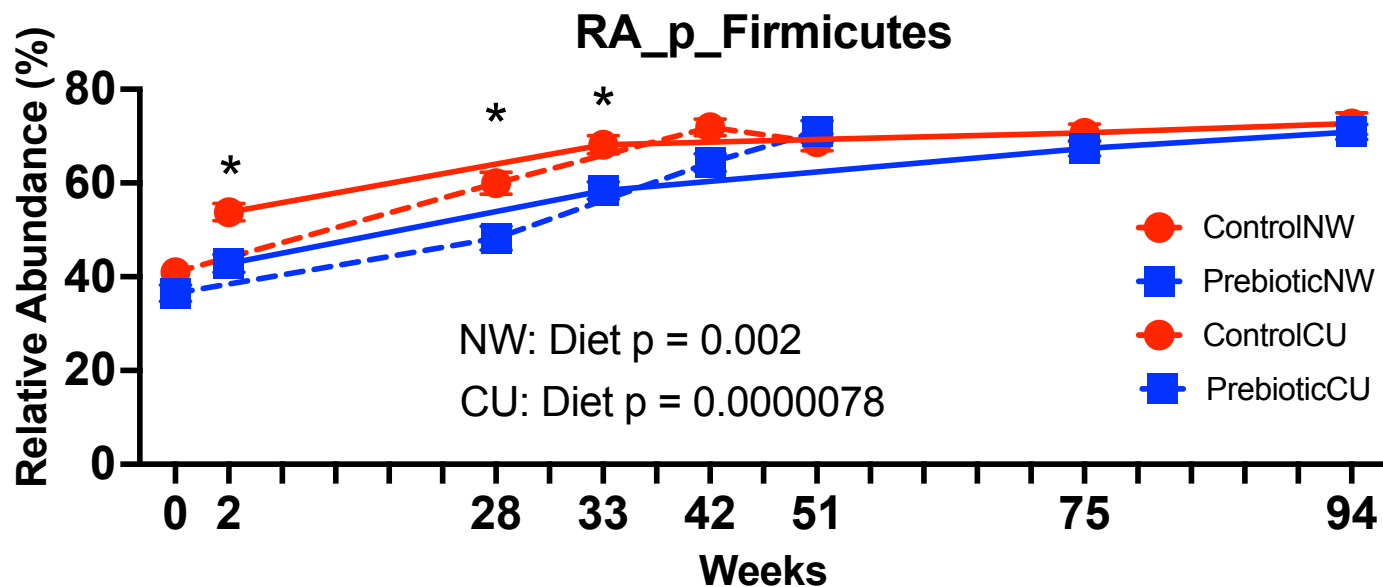

B)

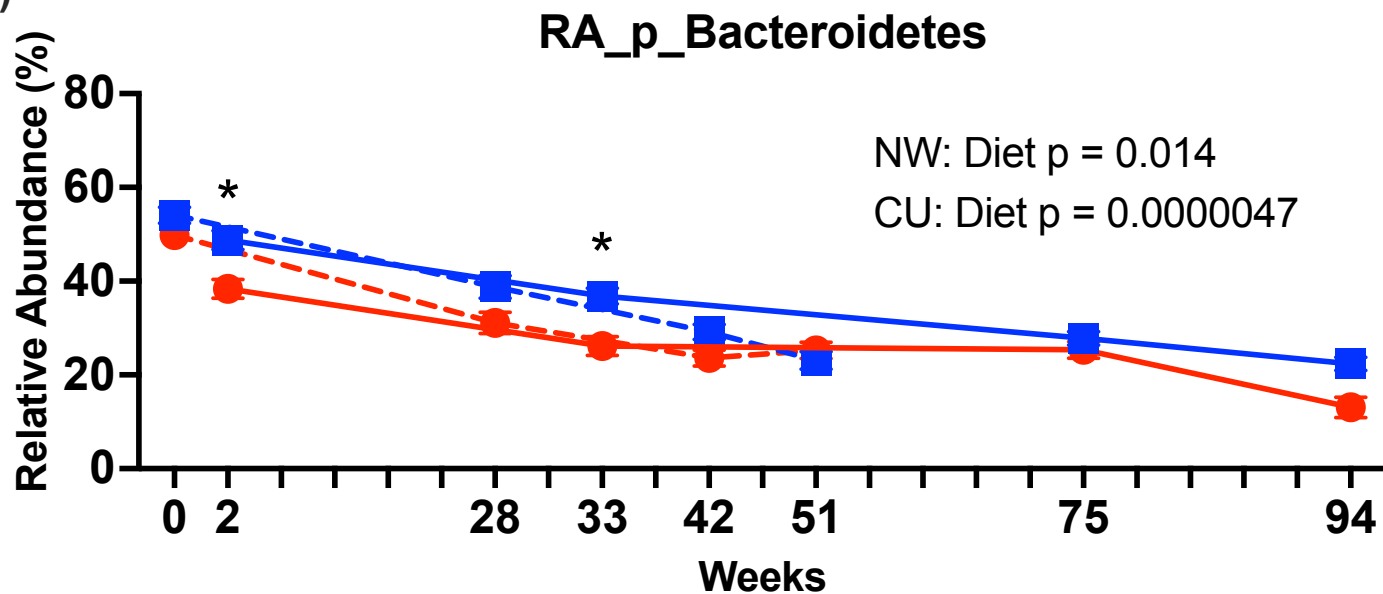

Supplement: Supplementary file 1 [file nutrients-16-01790-s001.zip › Figure S1.pdf]

RA\_g\_Muribaculaceae

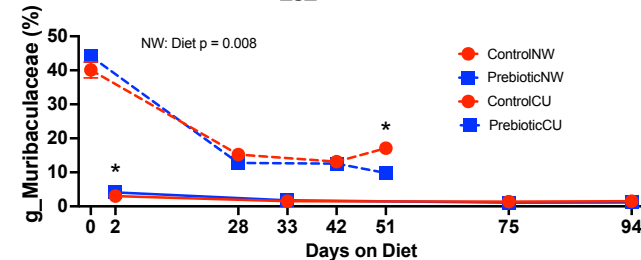

RA\_g\_Blautia

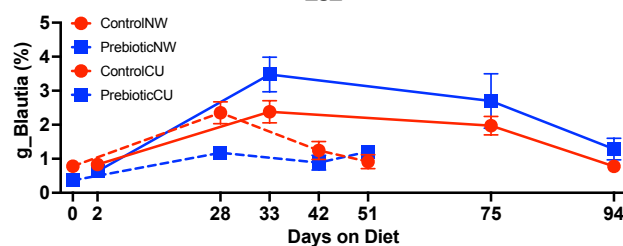

RA\_g\_RF39

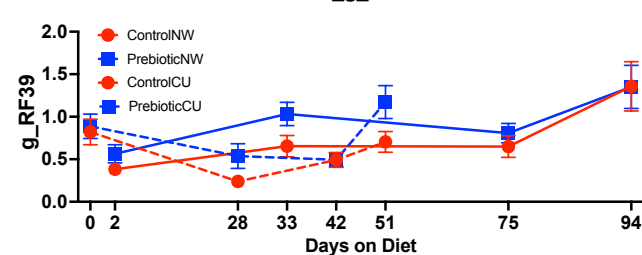

RA\_g\_Lachnoclostridium

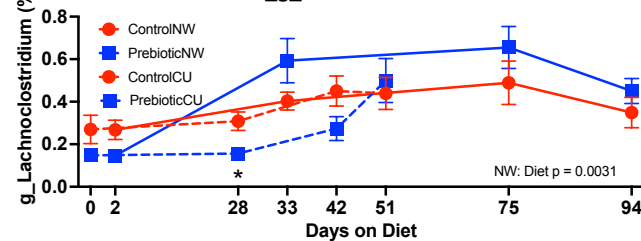

RA\_g\_A2

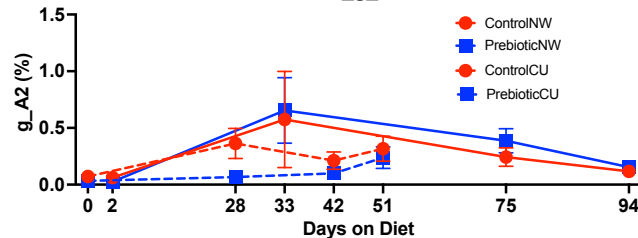

RA\_g\_Alistipes

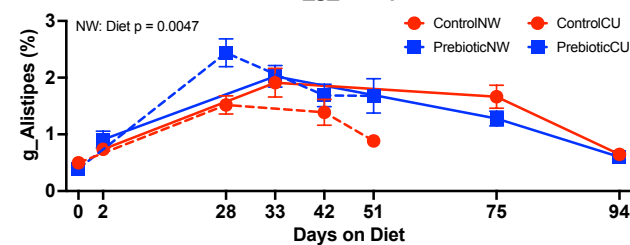

Supplement: Supplementary file 1 [file nutrients-16-01790-s001.zip › Figure S3.pdf]
